# Integrated 16S rDNA-Seq and metabolomics reveal seasonal dynamics of gut microbial–SCFA–immune crosstalk in diarrheic calves

**DOI:** 10.3389/fvets.2025.1615310

**Published:** 2025-07-11

**Authors:** Qianqian Wang, Qicheng Lu, Yaqin Tang, Qiuyan Li, Peiyun Gao, Shaoyang Pang, Wenju Zhang, Cunxi Nie, Junli Niu, Xiaoling Ma

**Affiliations:** ^1^College of Animal Science and Technology, Shihezi University, Shihezi, Xinjiang, China; ^2^Laboratory and Equipment Management Division, Shihezi University, Shihezi, Xinjiang, China

**Keywords:** lactating calves, diarrhea, seasons, intestinal permeability, rectal flora, short-chain fatty acids

## Abstract

**Introduction:**

Neonatal calf diarrhea incidence varies seasonally, increasing during climatic fluctuations. This study investigated body weight and size, immune function, intestinal permeability, rectal microbiota, and short-chain fatty acid (SCFA) levels in diarrheal and healthy lactating calves across seasons.

**Methods:**

Ten lactating healthy and ten diarrhea calves were selected in each season, blood and rectal contents samples were collected. Serum immunity, cytokines, and intestinal permeability markers were measured using kits. Rectal microbiota composition was analyzed via 16S rDNA amplicon sequencing, and SCFA profiles of rectal contents were characterized using targeted metabolomics.

**Results:**

Significantly higher levels of interleukin-1β (IL-1β), endotoxin (ET), and diamine oxidase (DAO) and lower levels of interleukin-10 (IL-10), immunoglobulin (IgA and IgG) levels were observed in diarrheic calves compared to those of healthy controls across all four seasons. Acetate and valeric acid concentrations were significantly lower in diarrheic calves in summer, autumn, and winter. In addition, diarrheic calves exhibited significantly reduced alpha diversity compared to than that of healthy controls, as indicated by lower Chao1, Observed_features, and Shannon indices. The relative abundances of *Escherichia-Shigella*, *Fusobacterium*, and *Peptostreptococcus* in the diarrheal calf group were significantly higher, while *Faecalibacterium* and *Bifidobacterium* were significantly lower. *Clostridium_sensu_stricto_1* content was positively correlated with ET and DAO and negatively correlated with IL-10 and IgA. *Escherichia-Shigella* was positively correlated with ET, DAO, IL-1β, and TNF-α, but negatively correlated with IL-10 and IgA. *Fusobacterium* was positively correlated with ET, DAO, and IL-1β.

**Discussion:**

In conclusion, seasonal factors have an influence on the indicators related to diarrhea in calves. Diarrheic lactating calves showed the characteristics of reduced immunity, increased inflammatory response, reduced rectal microbial diversity, and altered microbiota profiles and SCFA content, and these alterations were closely related to the occurrence of diarrhea in calves.

## 1 Introduction

Calf diarrhea is one of the most common diseases on dairy farms. Due to their immature digestive and nervous systems, calves are highly susceptible to diarrhea (1) and their weak self-regulatory abilities (2, 3). Calf diarrhea induces gut microbiota imbalance, barrier dysfunction, immune dysregulation, and metabolic disturbances (4), and in severe cases, this can lead to the death of calves. Diarrhea accounts for up to 57% of calf deaths (5), severely affecting their subsequent growth, development, and productive performance. This leads to substantial economic and productivity losses, thereby compromising the health and sustainable development of the livestock industry (6, 7).

Calf diarrhea has complex and varied etiologies, comprising bacterial infections (*Escherichia coli* and *Salmonella*), viral infections (*rotavirus and coronavirus*), and parasitic infections (*Cryptosporidium*), as well as improper feeding management practices (8). Weaning stress also influences diarrhea in calves by decreasing the immune function (9), making diarrhea the most common response to weaning stress (10). The incidence of diarrhea in newborn calves varies across seasons. Calf diarrhea is not confined to a specific season but tends to occur more frequently during periods of climate fluctuation, such as early spring, late summer, and early autumn (11). Additionally, calf diarrhea can occur year-round, with a higher prevalence in winter and early spring (12). These discrepancies may be attributed to regional climatic differences. Gut microbes are critical in maintaining animal health and inhibiting disease progression by influencing gastrointestinal conditions. Short-chain fatty acids (SCFAs), which are indirect nutrients produced by intestinal microbiota, primarily comprise acetate, propionate and butyrate, accounting for approximately 85% of the total SCFAs (13, 14). They are critical in protecting the intestinal mucosal barrier, promoting nutrient absorption, and inhibiting the growth of harmful bacteria (15). In addition, SCFAs can influence the colonization of calf intestinal flora, maintain intestinal barrier integrity, and enhance anti-inflammatory ability, thereby promoting the healthy growth of calves (16). SCFAs have anti-inflammatory properties, and SCFA-producing bacteria can reverse the gut microbiota imbalance and inhibit the secretion of pro-inflammatory cytokines (17). However, the relationship between body weight, immunity, inflammation, intestinal permeability, gut microbiota composition, and SCFA levels in diarrheic suckling calves across different seasons remains unclear.

This study aimed to analyze the body weight, serum immune indices, cytokine levels, and intestinal permeability indices of healthy and diarrheic suckling calves across different seasons. 16S rDNA high-throughput sequencing and targeted metabolomics were employed to compare the composition of the rectal microbiota and SCFA content in healthy and diarrheic suckling calves. Additionally, we investigated the relationships between serum immune markers, inflammatory factors, intestinal permeability indicators, rectal microbiota, SCFAs, and the incidence of calf diarrhea.

## 2 Materials and methods

### 2.1 Animals

Fecal samples were collected from Holstein lactating calves with similar body weight and day age (21 ± 3) at Shihezi dairy farm in April (spring), July (summer), October (autumn), and January (winter), respectively. The animal care protocol was approved by the Animal Welfare Committee of Shihezi University (Xinjiang, China) (Ethics No. A2023-313). One day before sample collection, the defecation status of Holstein suckling calves with comparable body weight and age in the farm was observed, and the calf fecal scores were performed according to the method described by Lee et al. (18). Calves with a fecal scores < 3 were categorized as the healthy, while those with a score ≥ 3 were classified as diarrheic. From the healthy and diarrheic calves, 10 individuals were randomly selected to form the healthy group (H) and the diarrheal group (D), respectively.

### 2.2 Sample collection

Ten healthy and ten diarrhea calves in each season were selected to collecting the blood and rectal contents samples. The blood samples collected from the jugular vein of calves were allowed to stand for 30 min, and the serum was separated by centrifugation at 3,000 rpm for 15 min and stored at −20°C. Fecal samples were collected from the rectum of calves, immediately transferred into enzyme-free and sterile freezing tubes, and rapidly frozen in liquid nitrogen. The samples were subsequently transported to the laboratory and stored in the refrigerator at −80°C.

### 2.3 Measurement of weight and body measurements

Body weight, height, slant length, and chest circumference were measured according to the Technical Specification for Measuring the Production Performance of Beef Cattle (NY/T 2660-2014).

### 2.4 Measurement of serum indicators

Serum samples for alanine aminotransferase (ALT), total protein (TP), albumin (ALB), globulin (GLB), menthyltransferase (AST), alkaline phosphatase (ALPU), urea (UN), blood glucose (GLU), triglycerides (TG), total cholesterol (TC), creatine kinase (CK), lactate dehydrogenase (LDH), uric anhydride (UA) were measured using an automatic biochemical analyzer (OLYMPUS AU5800; Olympus Corporation, Tokyo, Japan). Immunoglobulin M (IgM), immunoglobulin A (IgA), immunoglobulin G (IgG), tumor necrosis factor-α (TNF-α), interleukin-10 (IL-10), interleukin-1β (IL-1β), transforming growth factor-β (TGF-β), endotoxin (ET) and diamine oxidase (DAO) were measured using commercial ELISA kits (Shanghai Enzyme-linked Biotechnology Co., Ltd., China).

### 2.5 16S rDNA high-throughput sequencing and analysis

Total bacterial DNA was extracted from the cecal contents of 10 calves per group using a TIANamp Stool DNA kit (Tiangen, Beijing, China). DNA sequencing was performed on the Illumina MiSeq desktop sequencer (Illumina, CA, USA) by Macrogene Inc. (Majorbio, Shanghai, China), targeting the V3-V4 hypervariable region of the 16S rDNA gene with primer 5′-ACTCCTACGGGAGGCAGCA-3′ and 5′-GGACTACHVGGGTWTCTAAT-3′. High-quality sequences were processed using the QIIME2 software. The sequences were analyzed using UCLUST (version 7.1) and clustered into operational taxonomic units (OTUs) at a similarity level of 97%. Representative sequences were aligned against the Greengenes 13.5 database using PyNAST, and the default parameters were set using QIIME2. Alpha diversity analysis included an abundance-based coverage estimator, Chao1 richness estimate, and Shannon and Simpson diversity indices. Principal coordinate analysis (PCoA) was used to assess the UniFrac distances and sample clustering. Differential bacterial abundances among the groups were analyzed using linear discriminant analysis effect size (LEfSe). Functional prediction of the metabolic pathways in the gut flora was performed using PICRUSt2. Raw sequencing data were deposited in the NCBI Sequence Read Archive under accession number PRJNA1238067.

### 2.6 Targeted metabolomics of short-chain fatty acids

#### 2.6.1 Preparation of samples

An appropriate amount of calf rectal contents was placed in a 1.5 mL centrifuge tube, followed by the addition of 500 μL of water and 100 mg of glass beads. The sample was homogenized for 1 min and then centrifuged at 13,400×*g* for 10 min at 4°C to obtain the supernatant (200 μL). Subsequently, 100, 20, and 280 μL of 15% phosphoric acid, 4-methylpentanoic acid (375 μg/mL), and ether, respectively, were added to the supernatant.

#### 2.6.2 GC/MS analysis

##### 2.6.2.1 Gas chromatography conditions

The GC analysis was performed using a Trace 13 1 0 gas chromatograph (Thermo Fisher Scientific, USA). The GC was fitted with a capillary column Agilent HP-INNOWAX (30 m × 0.25 mm ID × 0.25 μm), and helium was used as the carrier gas at 1 mL/min. The injection was made in split mode at 10:1 with an injection volume of 1 μL and an injector temperature of 250°C. The temperature of the ion source and MS transfer line were 300 and 250°C, respectively. The column temperature was programmed to increase from an initial temperature of 90°C, followed by an increase to 120°C at 10°C/min, 150°C at 5°C/min, and finally to 250°C at 25°C/min, which was maintained for 2 min.

##### 2.6.2.2 Mass spectrum conditions

Mass spectrometric detection of metabolites was performed on an ISQ LT (Thermo Fisher Scientific, Waltham, MA, USA) in an electron impact ionization mode. The single-ion monitoring (SIM) mode was used with an electron energy of 70 eV. The calibration curve, linearity, correlation coefficients, limit of detection, and quantitation stability are presented in Supplementary Table S1.

### 2.7 Statistical analysis

Data were analyzed by two-way ANOVA using SPSS software (version 27.0), *p* < 0.05 was accepted as statistically significant. The repeated measures model contained fixed effects of treatment, season, and the interaction of treatment and season, and the random effect of calf identity. Significantly different blood markers, rectal microbiota (*q* < 0.05, relative abundance > 0.5%), and rectal content differential SCFA (*q* < 0.05) were selected for interaction analysis. Correlations with an absolute Spearman’s correlation coefficient ≥ 0.50 and a *q*-value <0.05 were considered significant. The Majorbio tools website^1^ was used to visualize the correlations.

## 3 Results

### 3.1 Weight and body size of healthy and diarrheic lactating calves in different seasons

No significant differences were observed between the groups and seasons in body size indices, such as body weight, body height, body slant length, and chest circumference of healthy and diarrhea-lactating calves in different seasons (*p* > 0.05; Table 1). However, lactating calves with diarrhea tended to have lower body weights (*p* = 0.0532 and *p* = 0.0622, respectively) during spring and winter.

**Table 1:** Weight and body size of healthy and diarrhea lactating calves in different seasons.

Item	Treatment		SEM	*p*-value			
	H	D		Trt	Season	Trt × Season	H × D
Body weight (kg)							
Spr.	48.82	47.64	6.62	0.263	0.351	0.541	0.053
Sum.	49.01	47.04	7.14				0.767
Aut.	48.93	46.91	6.59				0.123
Win.	48.64	47.24	5.99				0.062
Body height (cm)							
Spr.	79.73	79.23	8.49	0.322	0.201	0.410	0.192
Sum.	79.82	79.12	8.97				0.438
Aut.	80.00	80.01	9.38				0.373
Win.	81.08	80.88	10.88				0.764
Body oblique length (cm)							
Spr.	66.02	66.12	5.87	0.196	0.321	0.217	0.478
Sum.	66.18	66.28	6.10				0.378
Aut.	66.25	66.15	5.39				0.474
Win.	66.09	65.78	6.45				0.898
Heart girth (cm)							
Spr.	89.09	88.87	9.17	0.398	0.514	0.378	0.478
Sum.	89.18	88.78	9.66				0.676
Aut.	89.64	89.18	8.97				0.362
Win.	89.17	89.08	10.21				0.759

### 3.2 Blood biochemical indices of healthy and diarrheal lactating calves in different seasons

No significant differences were observed in the serum of healthy and diarrheic lactating calves in different seasons for alanine aminotransferase, total protein, albumin, globulin, urea, blood glucose, triglycerides, total cholesterol, creatine kinase, lactate dehydrogenase, and uric anhydride (*p* ˃ 0.05; Table 2). Serum levels of mentholatum transferase were significantly higher in diarrheic lactating calves than those in healthy calves in all seasons (*p* < 0.05). However, serum levels of alkaline phosphatase were significantly higher in healthy lactating calves than those in calves with diarrhea in spring, summer, and autumn (*p* < 0.05), and no significant difference was observed in winter (*p* > 0.05). None of the blood biochemical indices differed significantly between seasons (*p* > 0.05).

**Table 2:** Blood biochemical indices of healthy and diarrhea lactating calves in different seasons.

Item	Treatment		SEM	*p*-value			
	H	D		Trt	Season	Trt × Season	H × D
Alanine aminotransferase (U/L)							
Spr.	4.33	5.10	0.88	0.334	0.121	0.372	0.083
Sum.	5.50	5.21	0.76				0.848
Aut.	5.50	5.66	0.91				0.256
Win.	4.50	5.02	0.69				0.467
Total protein (g/L)							
Spr.	62.20	64.43	6.58	0.176	0.227	0.451	0.654
Sum.	60.70	57.66	9.45				0.891
Aut.	61.73	62.86	6.98				0.054
Win.	65.73	60.56	8.49				0.841
Albumin (g/L)							
Spr.	35.76	36.80	6.88	0.521	0.657	0.211	0.781
Sum.	36.35	36.01	6.08				0.673
Aut.	36.30	40.36	7.94				0.230
Win.	37.10	36.71	5.99				0.566
Globulin (g/L)							
Spr.	24.23	27.63	4.33	0.376	0.641	0.430	0.564
Sum.	24.85	21.50	3.86				0.567
Aut.	25.63	23.50	5.18				0.333
Win.	28.46	23.76	4.90				0.232
Menthyltransferase (U/L)							
Spr.	40.66^b^	53.14^a^	5.29	0.332	0.301	0.541	0.035
Sum.	41.50^b^	52.66^a^	6.10				0.023
Aut.	40.66^b^	54.88^a^	4.31				0.014
Win.	40.71^b^	48.33^a^	4.85				0.021
Alkaline phosphatase (U/L)							
Spr.	177.54^a^	140.33^b^	29.35	0.164	0.422	0.249	0.032
Sum.	153.51^a^	145.70^b^	30.26				0.023
Aut.	196.66^a^	150.66^b^	28.24				0.014
Win.	182.55	168.66	31.21				0.054
Urea (mmol/L)							
Spr.	2.80	3.53	0.49	0.565	0.451	0.540	0.098
Sum.	3.15	3.00	0.46				0.251
Aut.	2.50	3.93	0.57				0.751
Win.	2.46	2.53	0.39				0.250
Blood glucose (mmol/L)							
Spr.	5.60	5.36	0.88	0.149	0.263	0.216	0.551
Sum.	5.95	5.20	0.79				0.263
Aut.	6.05	5.30	0.92				0.457
Win.	5.50	5.56	0.98				0.658
Triglycerides (mmol/L)							
Spr.	0.46	0.46	0.02	0.152	0.541	0.355	0.265
Sum.	0.56	0.46	0.03				0.654
Aut.	0.54	0.40	0.02				0.443
Win.	0.46	0.48	0.03				0.325
Total cholesterol (mmol/L)							
Spr.	2.29	2.05	0.46	0.276	0.120	0.644	0.565
Sum.	2.47	2.55	0.52				0.265
Aut.	2.78	2.25	0.49				0.467
Win.	2.95	2.35	0.50				0.656
Creatine kinase (U/L)							
Spr.	223.33	186.66	31.25	0.532	0.540	0.232	0.254
Sum.	214.66	191.66	32.11				0.258
Aut.	242.66	153.66	29.98				0.654
Win.	232.66	149.66	30.54				0.456
Lactate dehydrogenase (U/L)							
Spr.	678.33	635.33	65.90	0.122	0.451	0.311	0.265
Sum.	665.33	633.32	66.23				0.325
Aut.	657.22	688.66	67.01				0.359
Win.	652.66	641.66	64.21				0.489
Uric anhydride (umol/L)							
Spr.	64.33	73.33	11.88	0.651	0.311	0.321	0.577
Sum.	67.32	72.66	13.01				0.257
Aut.	66.01	71.33	15.22				0.656
Win.	65.66	71.21	14.97				0.326

Different lowercase superscripts within a row indicate a significant difference (*p* < 0.05), while the same or no letter indicates a non-significant difference (*p* > 0.05).

### 3.3 Serum immunoglobulin levels in healthy and diarrheic lactating calves in different seasons

Serum IgA and IgG levels in healthy lactating calves were significantly higher than those in diarrheic calves in different seasons (*p* < 0.05; Table 3), while IgM levels were not significantly different between the two groups of calves (*p* > 0.05). In addition, none of the abovementioned immunological indices differed significantly between seasons (*p* > 0.05).

**Table 3:** Serum immunoglobulin levels in healthy and diarrheic lactating calves in different seasons.

Item	Treatment		SEM	*p*-value			
	H	D		Trt	Season	Trt × Season	H × D
IgA (μg/mL)							
Spr.	759.85^a^	589.59^b^	65.24	0.042	0.064	0.124	0.021
Sum.	783.41^a^	459.06^b^	68.02				0.002
Aut.	758.64^a^	508.16^b^	70.21				0.043
Win.	783.25^a^	574.12^b^	53.00				0.019
IgM (μg/mL)							
Spr.	1,771.92	1,759.09	231.87	0.135	0.316	0.155	0.063
Sum.	1,720.82	1,730.51	526.01				0.078
Aut.	1,745.11	1,709.29	266.56				0.083
Win.	1,715.22	1,699.86	565.30				0.075
IgG (g/L)							
Spr.	13.96^a^	11.64^b^	1.212	0.064	0.071	0.103	0.001
Sum.	13.54^a^	12.62^b^	2.004				0.043
Aut.	13.00^a^	12.05^b^	3.981				0.031
Win.	14.28^a^	12.46^b^	2.214				0.002

Different lowercase superscripts within a row indicate a significant difference (*p* < 0.05), while the same or no letter indicates a non-significant difference (*p* > 0.05).

### 3.4 Serum cytokine levels in healthy and diarrheic lactating calves in different seasons

The IL-1β content in diarrheic calves was significantly higher than that of healthy calves in all seasons. Similarly, the TNF-α levels were higher in diarrheic calves during spring, summer, and autumn (*p* < 0.05; Table 4). The serum levels of IL-10 were significantly higher in healthy calves than those in calves with diarrhea in spring, summer, and autumn (*p* < 0.05), but the difference was not significant in winter (*p* > 0.05). In addition, no significant difference was observed in the TGF-β content between healthy and diarrheic calves, as well as in each index between seasons (*p* > 0.05).

**Table 4:** Serum cytokines in healthy and diarrheic lactating calves in different seasons.

Item	Treatment		SEM	*p*-value			
	H	D		Trt	Season	Trt × Season	H × D
IL-1β (pg/mL)							
Spr.	244.73^b^	477.76^a^	89.541	0.027	0.081	0.151	0.003
Sum.	277.62^b^	414.98^a^	69.223				0.001
Aut.	237.56^b^	489.26^a^	99.414				0.042
Win.	247.30^b^	455.78^a^	56.241				0.051
IL-10 (pg/mL)							
Spr.	25.97^a^	18.76^b^	2.265	0.182	0.079	0.122	0.021
Sum.	29.01^a^	18.51^b^	5.321				0.033
Aut.	27.50^a^	16.59^b^	4.981				0.002
Win.	23.94	17.64	5.123				0.076
TNF-α (pg/mL)							
Spr.	160.79^b^	235.89^a^	56.322	0.065	0.097	0.231	0.022
Sum.	187.28^b^	260.95^a^	86.254				0.043
Aut.	183.48^b^	279.19^a^	56.211				0.003
Win.	195.72	235.39	68.570				0.064
TGF-β (pg/mL)							
Spr.	714.35	518.44	98.362	0.382	0.345	0.540	0.091
Sum.	754.89	493.09	87.656				0.063
Aut.	780.94	487.42	80.450				0.084
Win.	788.66	483.19	95.210				0.078

Different lowercase superscripts within a row indicate a significant difference (*p* < 0.05), while the same or no letter indicates a non-significant difference (*p* > 0.05).

### 3.5 Indicators of blood-intestinal permeability in healthy and diarrheic lactating calves in different seasons

Serum botulinum toxin (ET) and diamine oxidase (DAO) levels were significantly higher in diarrheic calves in different seasons than in healthy calves (*p* < 0.05), but the levels of each indicator were not significantly different between seasons (*p* > 0.05) (Table 5).

**Table 5:** Indicators of intestinal permeability of blood in healthy and diarrheic lactating calves in different seasons.

Item	Treatment		SEM	*p*-value			
	H	D		Trt	Season	Trt × Season	H × D
Endotoxin ET (EU/mL)							
Spr.	9.28^b^	15.92^a^	1.235	0.034	0.094	0.471	0.032
Sum.	9.54^b^	14.46^a^	1.987				0.041
Aut.	9.12^b^	15.23^a^	2.054				0.089
Win.	10.50^b^	14.91^a^	0.987				0.123
Diamine oxidase DAO (ng/mL)							
Spr.	4.29^b^	6.47^a^	2.012	0.046	0.074	0.132	0.001
Sum.	5.26^b^	6.61^a^	1.981				0.042
Aut.	4.47^b^	6.64^a^	2.312				0.042
Win.	4.98^b^	6.11^a^	3.021				0.034

Different lowercase superscripts within a row indicate a significant difference (*p* < 0.05), while the same or no letter indicates a non-significant difference (*p* > 0.05).

### 3.6 Multivariate statistical analysis of short-chain fatty acid standards

The relative standard deviation (RSD) values < 10% indicated good methodological stability and reliable data (Supplementary Figure S1A). The TIC profiles exhibited effective separation and well-defined peaks for all seven SCFAs and the internal standard (isocaproic acid), confirming the suitability of this method for analyzing SCFAs (Supplementary Figures S1B,C).

**Figure 1:**
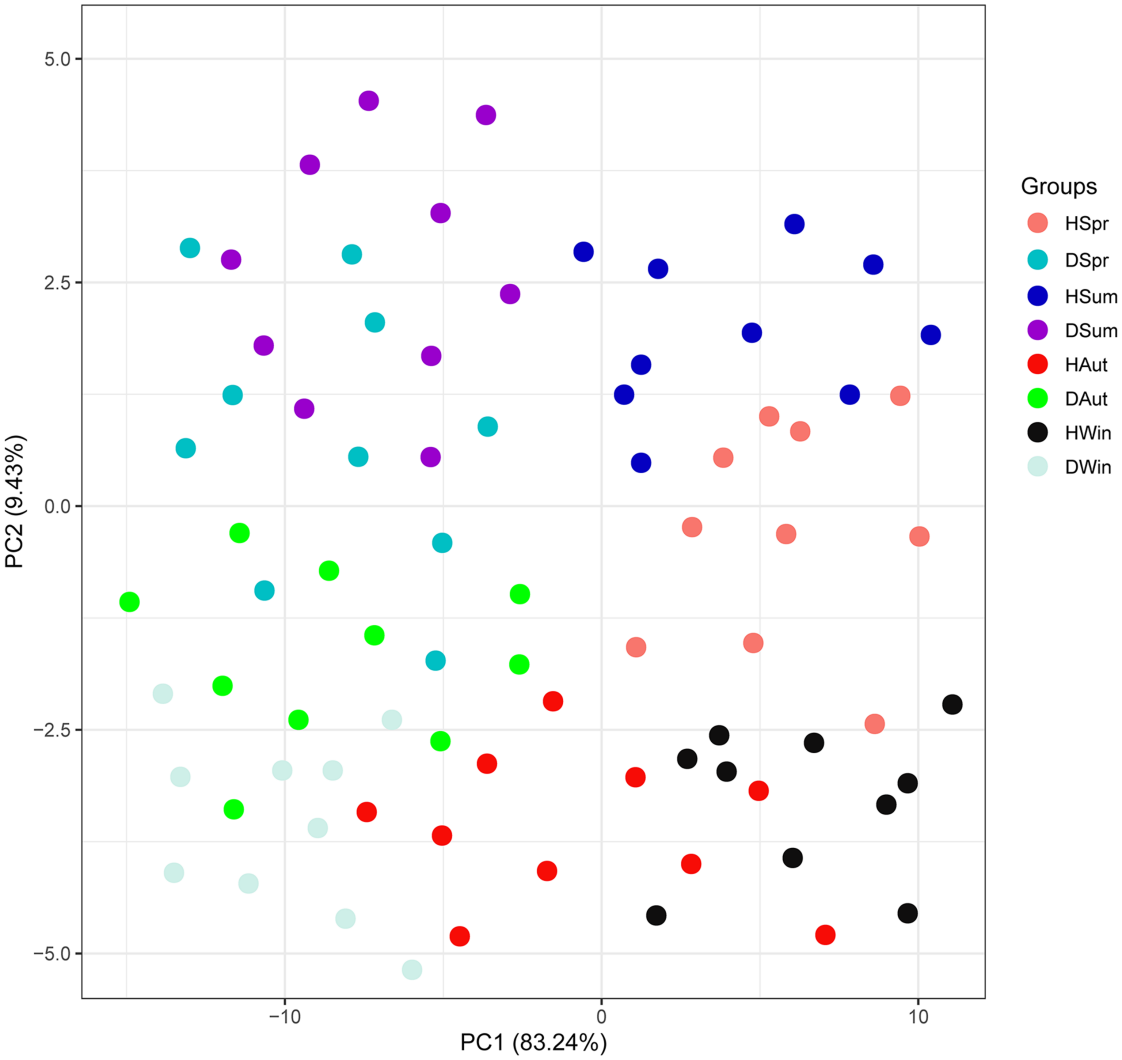
CA scores of rectal short-chain fatty acids in healthy and diarrheic calves in different seasons.

The proportions of the first principal component (PC1) and the second principal component (PC2) in the combined raw information were 83.24 and 9.43%, respectively, indicating a better distinction between healthy and diarrheic calves across different seasons. This suggests differences in SCFAs between the groups (Figure 1).

### 3.7 Comparative analysis of short-chain fatty acids

Table 6 indicates that rectal acetate levels in diarrheic calves were significantly lower than those in healthy calves in summer, autumn, and winter, as well as valeric acid levels in winter (*p* < 0.05); no significant differences were observed in other SCFAs between the groups (*p* > 0.05). In addition, the levels of acetate, propionate, valeric, and isovaleric acids differed significantly (*p* < 0.05) between seasons.

**Table 6:** Rectal short-chain fatty acid content of healthy and diarrheic calves in different seasons.

Item	Treatment		SEM	*p*-value			
	H	D		Trt	Season	Trt × Season	H × D
Acetate (μg/mL)							
Spr	216.30	152.99	20.21	0.076	0.031	0.530	0.441
Sum	121.10^a^	93.53^b^	18.54				0.034
Aut	282.19^a^	116.72^b^	26.32				0.022
Win	259.81^a^	133.61^b^	25.12				0.018
Propionic (μg/mL)							
Spr	87.61	52.89	6.21	0.162	0.025	0.381	0.215
Sum	58.96	22.75	5.32				0.364
Aut	70.19	26.16	6.63				0.651
Win	73.69	23.93	7.24				0.453
Isobutyric acid (μg/mL)							
Spr	11.46	4.77	1.03	0.265	0.043	0.152	0.451
Sum	4.22	1.33	0.22				0.167
Aut	11.49	2.46	0.89				0.163
Win	9.29	1.12	0.46				0.351
Butyrate (μg/mL)							
Spr	45.41	42.37	5.34	0.211	0.530	0.109	0.550
Sum	57.14	29.06	6.21				0.613
Aut	55.74	27.05	4.02				0.112
Win	45.69	32.92	4.56				0.243
Isovaleric acid (μg/mL)							
Spr	9.22	3.83	0.85	0.232	0.470	0.341	0.390
Sum	3.78	2.59	0.98				0.162
Aut	8.58	2.26	0.46				0.741
Win	7.04	1.37	0.94				0.248
Valeric acid (μg/mL)							
Spr	5.36	3.46	0.94	0.328	0.044	0.290	0.351
Sum	2.14	1.03	0.63				0.388
Aut	6.31	3.12	0.56				0.781
Win	2.72^a^	0.78^b^	0.54				0.011
Caproic acid (μg/mL)							
Spr	0.63	0.12	0.08	0.389	0.390	0.743	0.323
Sum	0.35	0.17	0.04				0.262
Aut	0.78	0.14	0.03				0.630
Win	0.25	0.23	0.02				0.143

Different lowercase superscripts within a row indicate a significant difference (*p* < 0.05), while the same or no letter indicates a non-significant difference (*p* > 0.05).

### 3.8 Rectal microbial changes

**Figure 2:**
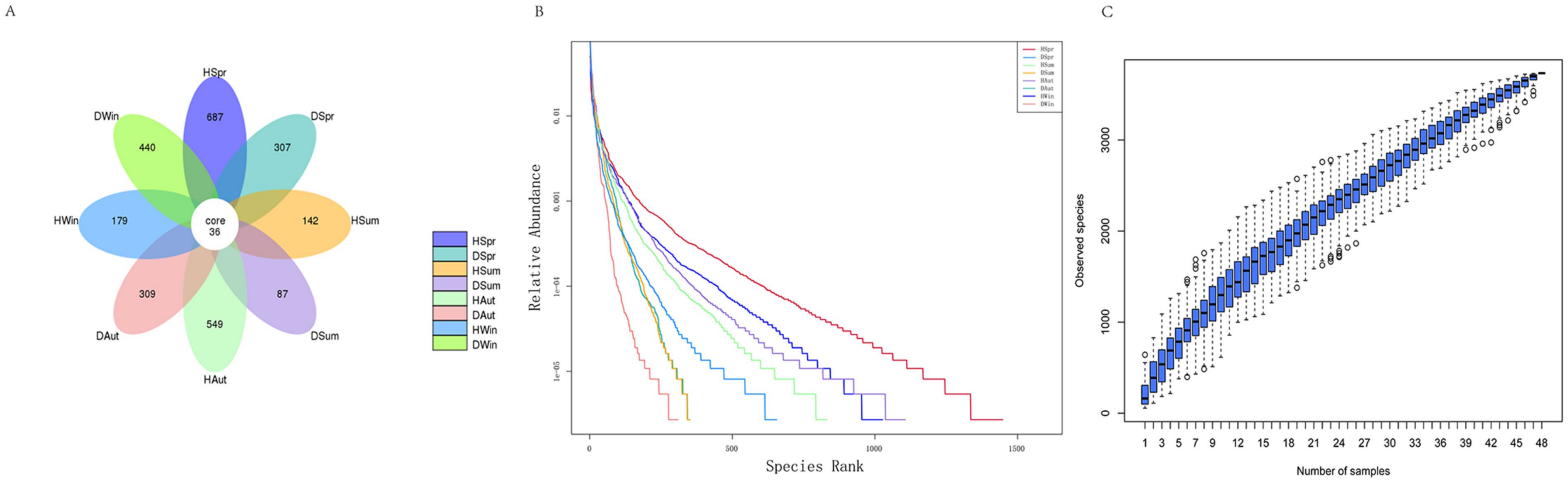
Rectal microbial Venn diagram, diversity rank-abundance clustering curves and species accumulation boxplot diversity in rectal of healthy and diarrhea calves. **(A)** Venn diagram. **(B)** Rank-abundance. **(C)** Species accumulation boxplot.

As shown in the Venn diagram (Figure 2A), 2,736 OTUs were identified, with a total of 36 OTUs in the healthy and diarrheal calf groups in the different seasons. A total of 1,157 and 1,143 OTUs were specific to the healthy and diarrheal calf groups, respectively. The number of OTUs specific to different seasons was 687 in the spring healthy group and 307 in the diarrheal group; 142 in the summer healthy group and 87 in the diarrheal group; 549 in the autumn healthy group and 309 in the diarrheal group; and 179 in the winter healthy group and 440 in the diarrheal group. The diversity rank-abundance curves demonstrated that the healthy calf group exhibited a broader extension along the horizontal axis, indicating higher species richness and even species distribution, with an overall flatter curve profile (Figure 2B). The species accumulation boxplot (Figure 2C) revealed that the boxplot positions gradually stabilized, confirming the adequacy of the sample size in this study.

To evaluate the ɑ-diversity of samples, the Chao1, Observed_features, Simpson, and Shannon indices were calculated in this experiment. Diarrheic calves exhibited significantly lower Chao1, Observed_features, and Shannon indices across different seasons than those of healthy calves (*p* < 0.05), whereas no significant difference was observed in the Simpson index between the two groups (*p* > 0.05). Additionally, none of the α-diversity indices showed significant seasonal variations (*p* > 0.05) (Table 7).

**Table 7:** Alpha diversity of rectal microorganisms in healthy and diarrheic calves.

Item	Treatment		SEM	*p*-value			
	H	D		Trt	Season	Trt × Season	H × D
Chao1							
Spr	239.36^a^	108.73^b^	25.748	0.032	0.084	0.121	0.027
Sum	296.58^a^	99.82^b^	18.974				0.033
Aut	269.83^a^	99.82^b^	16.872				0.034
Win	283.54^a^	86.52^b^	20.685				0.016
Observed_features							
Spr	226.17^a^	101.83^b^	28.191	0.021	0.130	0.425	0.021
Sum	206.20^a^	89.00^b^	20.571				0.017
Aut	286.00^a^	82.67^b^	15.222				0.022
Win	229.67^a^	83.50^b^	18.953				0.023
Shannon							
Spr	4.16^a^	3.29^b^	0.954	0.121	0.462	0.108	0.013
Sum	4.23^a^	3.79^b^	0.850				0.033
Aut	5.01^a^	3.29^b^	0.732				0.012
Win	4.77^a^	3.34^b^	0.801				0.014
Simpson							
Spr	0.96	0.91	0.081	0.342	0.144	0.477	0.771
Sum	0.89	0.82	0.094				0.830
Aut	0.93	0.87	0.075				0.243
Win	0.92	0.79	0.088				0.332

Different lowercase superscripts within a row indicate a significant difference (*p* < 0.05), while the same or no letter indicates a non-significant difference (*p* > 0.05).

**Figure 3:**
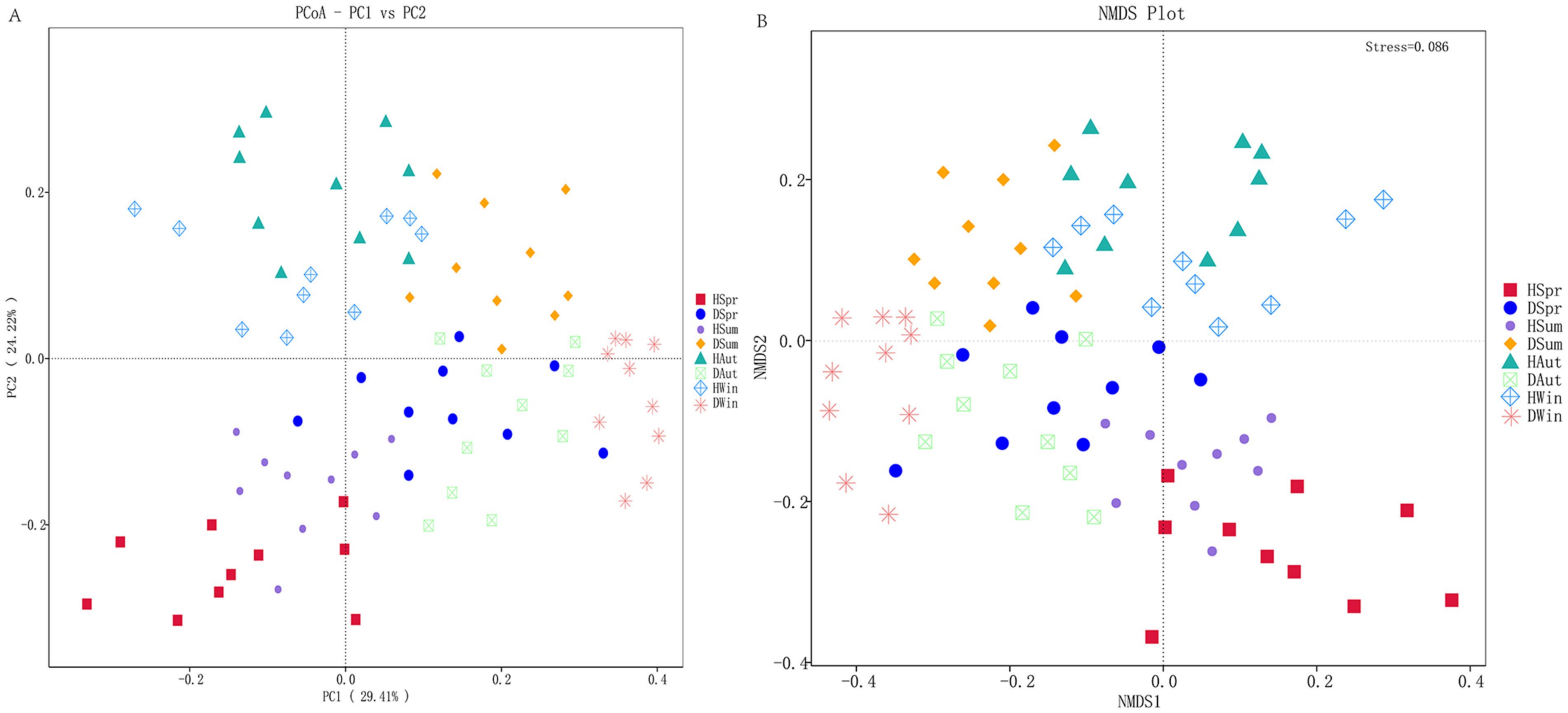
Principal coordinate analysis and non-metric multidimensional scaling of rectal microbes in healthy and diarrheic calves. **(A)** Principal coordinate analysis. **(B)** Non-metric multidimensional scaling.

The principal coordinates 1 and 2 in the PCOA accounted for 29.41 and 24.22% of the variation, respectively (Figure 3A). In the PCOA plot, samples from healthy calves clustered closely together across different seasons, indicating a high similarity in their microbial composition. In contrast, samples from diarrheic calves exhibited more dispersed distribution patterns, indicating considerable variability in the community structure among diarrheic individuals. The NMDS plot (Figure 3B) revealed that samples from healthy calves were tightly clustered regardless of the season, demonstrating minimal within-group variation and high structural consistency in their microbial communities. Conversely, samples from diarrheic calves exhibited a more scattered distribution, indicating significant heterogeneity in their microbial community structures.

**Figure 4:**
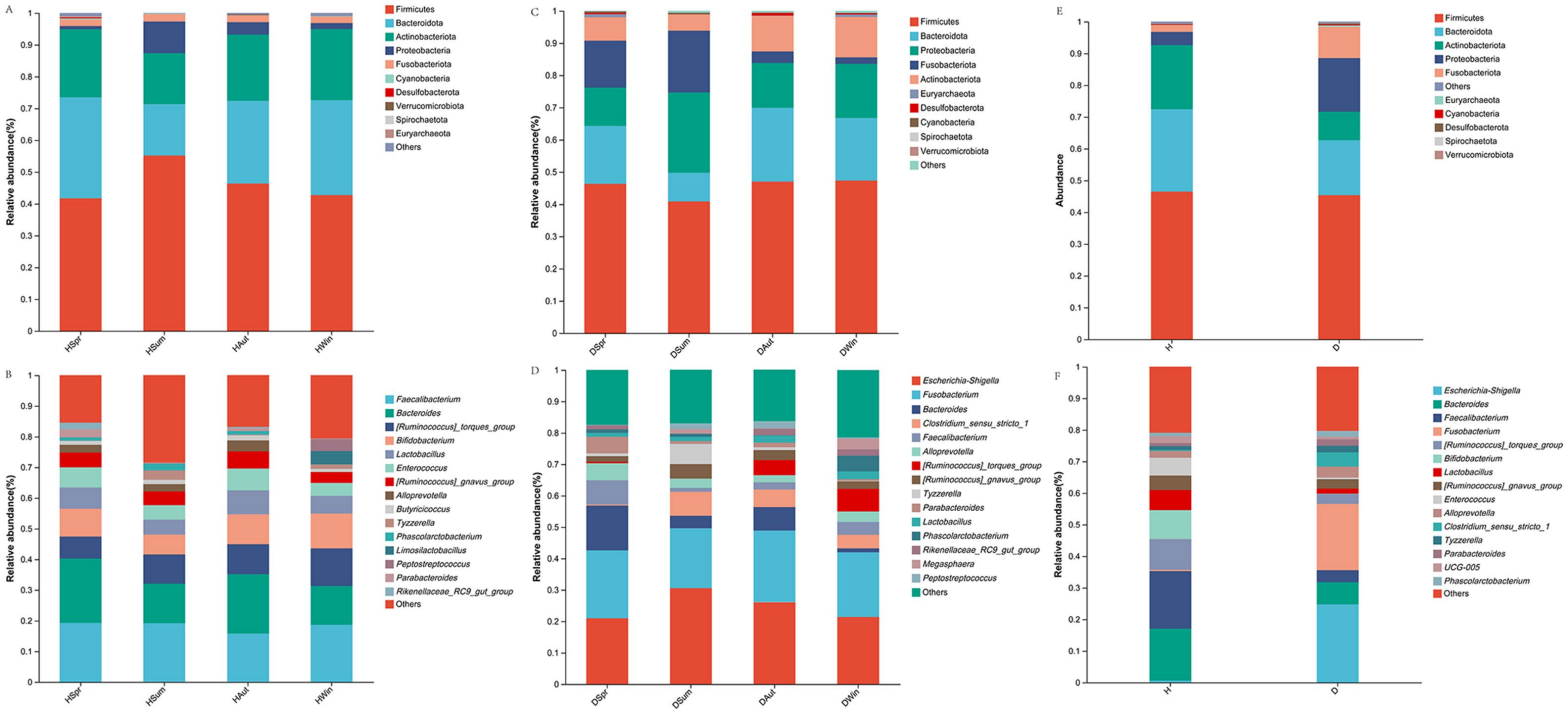
Rectal microbial community composition in healthy and diarrheic calves. **(A,C,E)** Microbiota phylum level composition. **(B,D,F)** Microbiota genus level composition.

Figures 4A,C show the top 10 phyla in healthy and diarrheic calves, respectively, across different seasons. The analysis results (Figure 4E) showed that Firmicutes, Bacteroidetes, Actinobacteria, Proteobacteria, and Fusobacteria were the major phyla in both calf groups. Figures 4B,D show the top 15 genera in the healthy and diarrheal calf groups across different seasons, respectively. *Escherichia-Shigella*, *Bacteroides*, and *Faecalibacterium* were the dominant genera in the healthy and diarrheic calf groups (Figure 4F).

**Figure 5:**
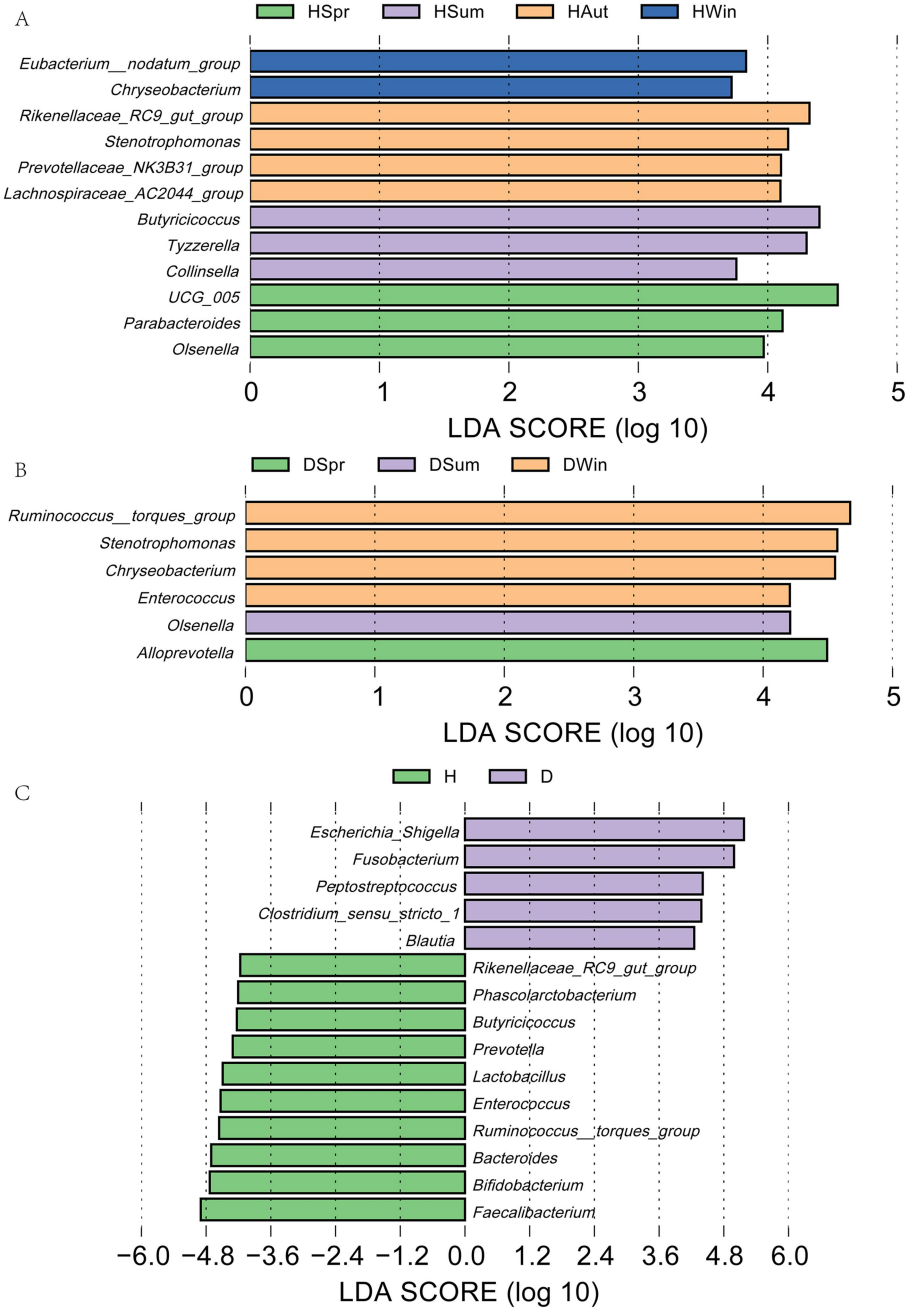
LEfSe analysis of rectal microflora in healthy and diarrheic calves. LDA discriminant plot with vertical coordinates indicating microorganisms with significant differences in multiple groups and horizontal coordinates (LDA scores) indicating species abundance. **(A)** LDA plots of healthy calves in different seasons; **(B)** LDA plots of calves with diarrhea in different seasons; **(C)** LDA plots of healthy and diarrheic calves.

In healthy calves, *UCG_005*, *Parabacteroides*, and *Olsenella* exhibited higher relative abundances in spring. *Butyricoccus* and *Tyzzerella* were more prevalent in the summer. *Rikenellaceae_RC9_*gut*_group*and *Stenotrophomonas* dominated in autumn, whereas *Eubacterium_nodatum_group* and *Chryseobacterium* were enriched in winter (Figure 5A). Conversely, diarrheic calves exhibited distinct seasonal patterns: *Alloprevotella* was predominant in spring, *Olsenella* in summer, and *Ruminococcus_torques_group* with *Stenotrophomonas* in winter (Figure 5B). LEfSe analysis further confirmed significant differences in the rectal flora composition between the groups (Figure 5C). The diarrheic group demonstrated considerable enrichment of *Escherichia-Shigella*, *Fusobacterium*, *Peptostreptococcus*, and *Clostridium_sensu_stricto_1* (*p* < 0.05). In contrast, healthy calves exhibited significantly higher abundances of *Faecalibacterium*, *Bifidobacterium*, *Bacteroides*, *Ruminococcus_torques_group*, *Enterococcus*, and *Lactobacillus* (*p* < 0.05).

**Figure 6:**
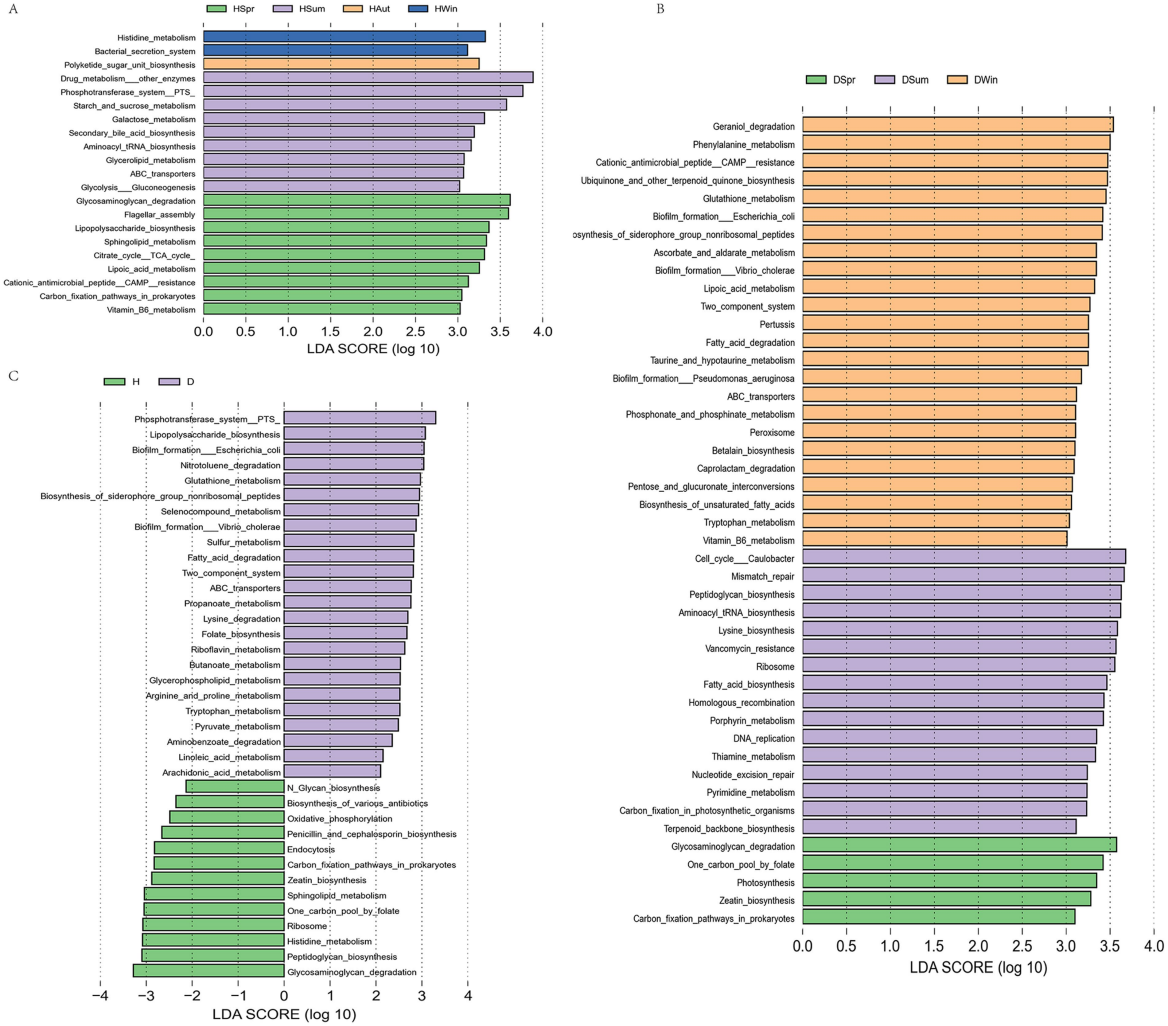
Functional prediction analysis of healthy and diarrheic rectal flora [KEGG pathway level 3 level **(C)**]. **(A)** LDA plots of healthy calves in different seasons; **(B)** LDA plots of calves with diarrhea in different seasons; **(C)** LDA plots of healthy and diarrheic calves.

To further investigate the potential functions of the calf rectal microbiota, we performed functional prediction analysis using the PICRUSt2 software based on the KEGG database. At KEGG pathway level 3, the healthy calf group showed upregulation of nine pathways in spring, including glycosaminoglycan degradation, flagellar assembly, and lipopolysaccharide biosynthesis. Summer was characterized by the enrichment of nine pathways, including drug metabolism—other enzymes, phosphotransferase system (PTS), and starch and sucrose metabolism. In autumn and winter, polyketide sugar unit biosynthesis, histidine metabolism, and bacterial secretion systems were upregulated (Figure 6A). In contrast, the diarrheic calf group exhibited the upregulation of four pathways in spring, including glycosaminoglycan degradation and a folate-mediated one-carbon pool. Sixteen pathways were enriched during summer, including the Caulobacter cell cycle, mismatch repair, and peptidoglycan biosynthesis. During winter, 24 pathways were upregulated, including geraniol degradation, phenylalanine metabolism, and cationic antimicrobial peptide (CAMP) resistance (Figure 6B). Twenty-four pathways were upregulated in the diarrheic group, including PTS, lipopolysaccharide biosynthesis, *Escherichia coli* biofilm formation, nitrotoluene degradation, glutathione metabolism, and fatty acid metabolism. The healthy group exhibited enrichment in 13 pathways, which included N-glycan biosynthesis, biosynthesis of various antibiotics, and oxidative phosphorylation (Figure 6C).

### 3.9 Correlation analysis of blood immunity, inflammation and intestinal permeability indicators, rectal short-chain fatty acids, and rectal flora

**Figure 7:**
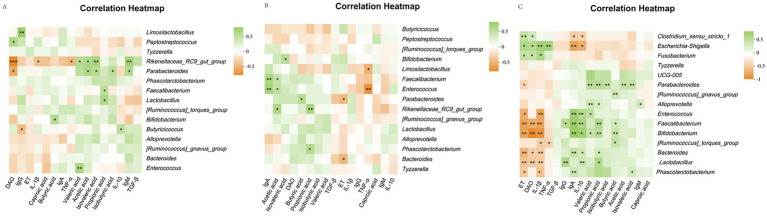
Heatmap of correlation analysis between serum immunity, inflammation and intestinal permeability differential indicators, rectal differential flora and differential short chain fatty acids in calves. **(A)** Heatmap for correlation analysis between indicators of healthy calves; **(B)** Heatmap for correlation analysis between indicators of diarrhea calves; **(C)** Heatmap for correlation analysis between indicators of healthy and diarrhea calves.

The correlation analysis showed that the rectal *Clostridium_sensu_stricto_1* content in calves was positively correlated with ET and DAO (*p* < 0.05) and negatively correlated with IL-10 and IgA (*p* < 0.05). The relative abundance of *Escherichia-Shigella* was positively correlated with ET, DAO, IL-1β, and TNF-α (*p* < 0.05) and negatively correlated with IL-10 and IgA (*p* < 0.05). *Fusobacterium* was positively correlated with ET, DAO, and IL-1β (*p* < 0.05). *Parabacteroides* levels were positively correlated with the intestinal permeability indicators isobutyric, isovaleric, acetate, propionate, and valeric acids (*p* < 0.05) and negatively correlated with ET (*p* < 0.05). *Alloprevotella* was positively correlated with IgM, valeric acid, and propionate levels (*p* < 0.05). The relative abundance of *Enterococcus* was positively correlated with IL-10, IgA, and valeric acid contents (*p* < 0.05) and negatively correlated with ET and IL-1β (*p* < 0.05). *Faecalibacterium* was positively correlated with butyrate, propionate, IL-10, IgA, and IgG (*p* < 0.05) and negatively correlated with IL-1β, ET, and DAO (*p* < 0.05). *Bifidobacterium* was positively correlated with butyrate, propionate, valeric acid, IL-10, and IgA contents (*p* < 0.05) and negatively correlated with IL-1β, ET, and DAO (*p* < 0.05). The relative abundance of the *[Ruminococcus]_torques_group* was positively correlated with butyrate content (*p* < 0.05) and negatively correlated with IL-1β and TNF-α (*p* < 0.05). The relative abundance of *Bacteroides* was positively correlated with propionate, IL-10, and IgA contents (*p* < 0.05) and negatively correlated with IL-1β, ET, and DAO (*p* < 0.05). *Lactobacillus* was positively correlated with propionate, IL-10, and IgG contents (*p* < 0.05) and negatively correlated with IL-1β, ET, and DAO (*p* < 0.05). The relative abundance of *Phascolarctobacterium* was positively correlated with isovaleric acid and IgA contents (*p* < 0.05) and negatively correlated with the intestinal permeability indices ET and IL-1β (*p* < 0.05) (Figure 7).

## 4 Discussion

Calf body weight and body measurements are important parameters for assessing overall health. Diarrhea often results in poor nutrient absorption; therefore, body weight measurements can be used as an indirect indicator to assess the nutritional status of calves, consequently providing a basis for adjusting feeding management programs. In the present study, we observed that calves with diarrhea tended to lose weight in both spring and winter. This finding is consistent with previous findings indicating that diarrhea in calves significantly affects digestibility, thus adversely affecting weight gain (19).

Immunoglobulins play a direct role in humoral immunity, and their concentrations partially indicate the immune ability of animals, which is crucial for the host to resist attacks by pathogenic microorganisms. IgG, the predominant antibody in serum (accounting for approximately 75% of total immunoglobulins), is a key indicator of the innate immunity response and the ability of an organism to combat infection (20). IgA, the second most abundant serum immunoglobulin, is a key element of the mucosal defense system. It inhibits microbial adhesion and constitutes the primary barrier against pathogenic invasion. Serum IgG levels indicate immune status, whereas IgM is related to anti-inflammation. Higher IgG and IgM levels typically indicate better immune function (21). The results of this study demonstrated that calves with diarrhea had significantly lower serum IgG and IgA levels than those of healthy calves.

Cytokines play a crucial role in livestock diarrhea by regulating immune responses and influencing intestinal physiological functions. Interleukin-1β (IL-1β), a key pro-inflammatory cytokine, is essential for immune response and pathological inflammation (22). IL-10, secreted by Th2 cells, is a vital regulator of immune homeostasis by controlling the chronic stimulation of the gut microbiota and food antigens (23). IL-10 is a potent anti-inflammatory cytokine that terminates inflammatory responses. Tumor necrosis factor-α (TNF-α) is a pleiotropic cytokine that acts on multiple cell types. It is a master regulator of the inflammatory response and contributes to the pathogenesis of various inflammatory and autoimmune diseases (24). Dysregulated or excessive activation of TNF-α signaling is associated with chronic inflammation, potentially leading to pathological complications such as autoimmune disorders (25). Chen et al. (26) reported that clinically diarrheic piglets showed significantly increased levels of pro-inflammatory cytokines (TNF-α and IL-1β) in jejunal tissues than those of healthy pigs, while the anti-inflammatory cytokine IL-10 exhibited a decreasing trend (though not statistically significant). Consistent with the above findings, the present study found that diarrheic calves had increased serum IL-1β and TNF-α levels but reduced IL-10 concentrations than those of healthy calves. Gut microbiota dysbiosis may influence increased pro-inflammatory cytokines, subsequently suppressing immunoglobulin synthesis, which is a plausible explanation for the decreased immunoglobulin levels observed in diarrheic calves (27). This mechanistic link further explains why the diarrheic calves in our study exhibited significantly lower serum IgG and IgA activities than those of healthy calves.

Impairment of the intestinal barrier function increases disease susceptibility in animals, making the maintenance of gut barrier integrity crucial for animal health. Serum diamine oxidase (DAO) is a key biomarker for evaluating gastrointestinal integrity. As an intracellular enzyme that is highly active in the upper villi of the small intestinal mucosa, DAO protects the intestinal epithelium by regulating the intracellular ion balance to promote cellular repair (28). Endotoxin (ET), a component of Gram-negative bacterial cell walls, can induce cellular inflammation and is widely used to establish inflammatory cell models (29). DAO functions as a marker enzyme in the cytoplasm of the intestinal epithelial cells. When the intestinal barrier function is compromised and mucosal permeability increases, substantial amounts of DAO and ET enter the bloodstream. Consequently, serum DAO activity and ET levels directly indicate the integrity of the intestinal barrier. Serum DAO activity in diarrheic calves is lower than that in control calves (30). In addition, increased intestinal permeability in animals, including calves, results in the entry of ET into circulation, subsequently triggering a systemic inflammatory response and liver injury. ET increases the secretion of pro-inflammatory factors by activating inflammatory signaling pathways and affects liver function through the gut-liver axis (31). This explains the increased serum levels of pro-inflammatory factors in calves with diarrhea in the present study.

SCFAs are the end products of the gut microbial fermentation of indigestible dietary fibers and serve as energy substrates for the host while protecting the intestinal mucosal barrier and suppressing gut inflammation (32). Major SCFAs, including acetate, propionate, butyrate, and isovalerate, play pivotal roles in the regulation of gut microbiota composition, immunity, metabolism, and the improvement of intestinal function (33, 34). Acetate (primarily produced by *Lactobacillus*) acidifies the intestinal environment to promote microbial balance, while excess acetate upregulates pro-inflammatory cytokines (IL-1β and IL-6), potentially triggering inflammation (35). Propionate can stimulate anti-inflammatory factors and upregulate claudin-1 expression to strengthen intestinal barrier integrity. Butyrate is the primary energy source for colonocytes and is critical for maintaining the gut barrier function (36). The results of this study revealed a significant reduction in rectal acetate and valeric acid levels in calves with diarrhea.

The intestinal flora, characterized by its abundance, diverse species, and complex structure, is a crucial component of the intestinal microecosystem. Interdependence and organizational crosstalk between intestinal flora lead to metabolic dysregulation and inflammation (37, 38).

The genus *Faecalibacterium*, which belongs to Firmicutes, is a symbiotic bacterium that colonizes the gastrointestinal tract of mammals and is one of the predominant bacterial taxa in the gut microbiota of healthy hosts (39). Faecalibacterium is a primary producer of butyrate and plays a crucial role in intestinal microecology (40). In addition to serving as an important energetic substance, butyrate is critical in immunomodulation, maintenance of the intestinal epithelial mucosal barrier function, and intestinal homeostasis (41, 42). Butyrate can regulate intestinal peristalsis and hormone secretion by activating G protein-coupled receptors, thus maintaining intestinal epithelial barrier function (43).

Clostridium_sensu_stricyto_1 is a highly diverse group comprising multiple species with distinct functional roles in the intestinal ecosystem. Although particular Clostridium species function as beneficial commensals that contribute to gut homeostasis, others exhibit pathogenic potential. Supplementation with Clostridium_sensu_stricyto_1 significantly increases IgA and IgG levels in calf serum (44), decreases DAO and D-lactate levels in piglet serum (45), and induces intestinal IL-10 production (46). Contrary to these findings, the present study found that the relative abundance of Clostridium_sensu_stricyto_1 was significantly higher in the diarrheal calf group than that in the healthy calf group, was positively correlated with ET and DAO, and negatively correlated with IL-10 and IgA. These phenomena may result from complex interactions among multiple factors. Certain pathogenic species within the Clostridium group may produce toxins or induce inflammatory responses, thereby compromising intestinal barrier integrity and disrupting immune homeostasis. However, a combination of factors, including gut barrier dysfunction, exacerbation of the inflammatory response, imbalance in the structure of the intestinal microbial community, developmental stage of the calf immune system, and stress response may also have contributed to this phenomenon.

*Escherichia coli*, a Gram-negative, parthenogenetic anaerobic bacterium of the Enterobacteriaceae family, has various pathogenic mechanisms, such as adhesion, invasion, and toxin secretion, which contribute to the induction of intestinal infections (47). The genus *Shigella* also belongs to the Enterobacteriaceae family and is phylogenetically closely related to the *Escherichiaceae* genus, with some strains sharing higher genetic homology with certain *E. coli* strains. *Shigella* is a major pathogen that causes bacillary dysentery, and its pathogenic mechanism involves multiple pathways, including evasion of host immune surveillance and induction of intestinal inflammation (48). Pathogenic *E. coli* can trigger severe intestinal inflammatory responses, leading to increased intestinal permeability and high levels of inflammatory cytokines (49). Consistent with these findings, we observed that the relative abundance of *Escherichia-Shigella* in the rectum of diarrheic calves was significantly higher than that in healthy calves. Moreover, it was positively correlated with ET, DAO, IL-1β, and TNF-α while negatively correlated with IL-10 and IgA.

The genus *Ruminococcus torques* is an important butyrate-producing bacterial group in gut microbiota. Butyrate, a SCFA, is crucial in maintaining intestinal homeostasis and regulating immune responses. *Ruminococcus torques* exhibit anti-inflammatory properties and inhibit the production of pro-inflammatory cytokines by modulating immune responses. Additionally, the abundance of this genus was positively correlated with butyrate levels (50). Consistent with these results, the present study revealed that the relative abundance of *Ruminococcus_torques_group* in the rectum of healthy calves was significantly higher than that in calves with diarrhea. Additionally, its relative abundance was positively correlated with butyrate levels and negatively correlated with pro-inflammatory cytokines IL-1β and TNF-α, suggesting its potential beneficial role in maintaining intestinal health in calves.

*Fusobacterium* is a key component of plaque biofilms and is essential in the development of periodontal diseases. *Fusobacterium* can activate host cells through surface adhesins and invasins, induce inflammatory responses, and promote the expression of pro-inflammatory cytokines. Additionally, it can disrupt tight junctions in intestinal epithelial cells, increasing intestinal permeability and leading to increased serum levels of ET and DAO (51). Consistent with the above findings, our study observed that the relative abundance of *Fusobacterium* was significantly higher in diarrheic calves than in healthy calves. Moreover, its relative abundance was positively correlated with ET, DAO, and IL-1β.

*Faecalibacterium*, a group of strict anaerobes belonging to the phylum Firmicutes, is a vital component of the gut microbiota in humans and animals. *Faecalibacterium* produces SCFAs such as butyrate and propionate. Butyrate is important in enhancing intestinal barrier integrity by regulating the expression of tight junction proteins in intestinal epithelial cells, thereby reducing intestinal permeability (52). Previous studies have confirmed a positive correlation between Faecalibacterium and the anti-inflammatory cytokine IL-10 as well as immunoglobulin IgA and IgG (53). Our findings further support these observations, demonstrating that the relative abundance of *Faecalibacterium* in the rectum of healthy calves was significantly higher than that in calves with diarrhea. Moreover, its abundance was positively correlated with butyrate, propionate, IL-10, IgA, and IgG while negatively correlated with IL-1β, ET, and DAO. These results highlight the critical role of *Faecalibacterium* in maintaining intestinal health and immune homeostasis in calves.

*Bifidobacterium* can perform various functions that are beneficial for host health. For example, exopolysaccharide (EPS) produced by *Bifidobacterium* can significantly modulate host immune function by promoting the secretion of the anti-inflammatory cytokine IL-10 while inhibiting the expression of the pro-inflammatory cytokine TNF-α (54). In addition, *Bifidobacterium* enhances the concentration of SCFAs in the gut, considerably improving the composition and functionality of the gut microbiota and strengthening the integrity of the intestinal barrier (55). Consistent with these findings, the present study revealed that the abundance of *Bifidobacterium* was significantly higher in healthy calves than in those with diarrhea. Furthermore, its abundance was positively correlated with butyrate, propionate, valerate, IL-10, and IgA while negatively correlated with IL-1β, ET, and DAO.

This study systematically evaluated the differences in body measurements, blood biochemical parameters, serum immune markers, cytokine levels, intestinal permeability, rectal microbiota composition, and SCFA profiles between diarrheic and healthy suckling calves across different seasons. We also preliminarily explored the relationships among blood biochemical indicators, gut microbiota, and SCFAs, providing valuable insights into the effect of diarrhea on calf health. However, this study has certain limitations. First, all the experimental samples were collected from a single farm, which may limit the generalizability and representativeness of the findings. Diarrhea is a multifactorial disease, and its different etiologies may lead to distinct patterns of gut microbial and metabolic disturbances. Therefore, future studies should consider the influence of causative agents. To gain a comprehensive understanding of the mechanisms underlying diarrhea, future studies should employ experimental interventions to validate the causal relationships between gut microbial and metabolic changes and diarrhea. Additionally, integrating multi-omics approaches, such as metagenomics, metabolomics, and transcriptomics, can help comprehensively elucidate the gene expression and metabolic functions of the calf gut microbiota, as well as host immune responses. These advancements can provide a basis for developing more effective strategies for the prevention and treatment of diarrhea.

## 5 Conclusion

The present study showed that diarrhea in calves significantly affected their serum immunity indexes, inflammatory factor levels and intestinal microecological structure. Diarrheic calves were generally characterized by decreased immune parameters, increased inflammatory factors and increased intestinal permeability. Regarding intestinal flora, diarrheic calves showed reduced rectal flora diversity and altered microbiota profiles, with increased abundance of specific pathogenic bacteria and decreased levels of some beneficial bacteria and short-chain fatty acids. In addition, we observed that seasonal factors may have an effect on some indicators, such as significant differences in alkaline phosphatase, TNF-α and IL-10 levels between healthy and diarrheal groups in spring, summer and autumn, but not in winter, and significantly higher levels of acetate in healthy calves than diarrheal groups in summer, autumn and winter. Additionally, these results provide a rationale for developing novel prevention and treatment strategies targeting microbial modulation to manage diarrheal conditions in calves.

## Data Availability

The datasets presented in this study can be found in online repositories. The names of the repository/repositories and accession number(s) can be found in the article/Supplementary material.

## Footnotes

^1^ https://doi.org/10.1002/imt2.12

## Supplementary material

FIGURE S1 Stability of short-chain fatty acids in QC samples, mixed sample and sample TIC plots. **(A)** Stability of short-chain fatty acids in QC samples; **(B)** mixed-label TIC plot; **(C)** sample TIC plot.
