# Supplementary figures and images for: Integrated 16S rDNA-Seq and metabolomics reveal seasonal dynamics of gut microbial–SCFA–immune crosstalk in diarrheic calves
Source: Front Vet Sci. 2025 Jul 11;12:1615310. doi: 10.3389/fvets.2025.1615310 (PMC12290471; doi:10.3389/fvets.2025.1615310)

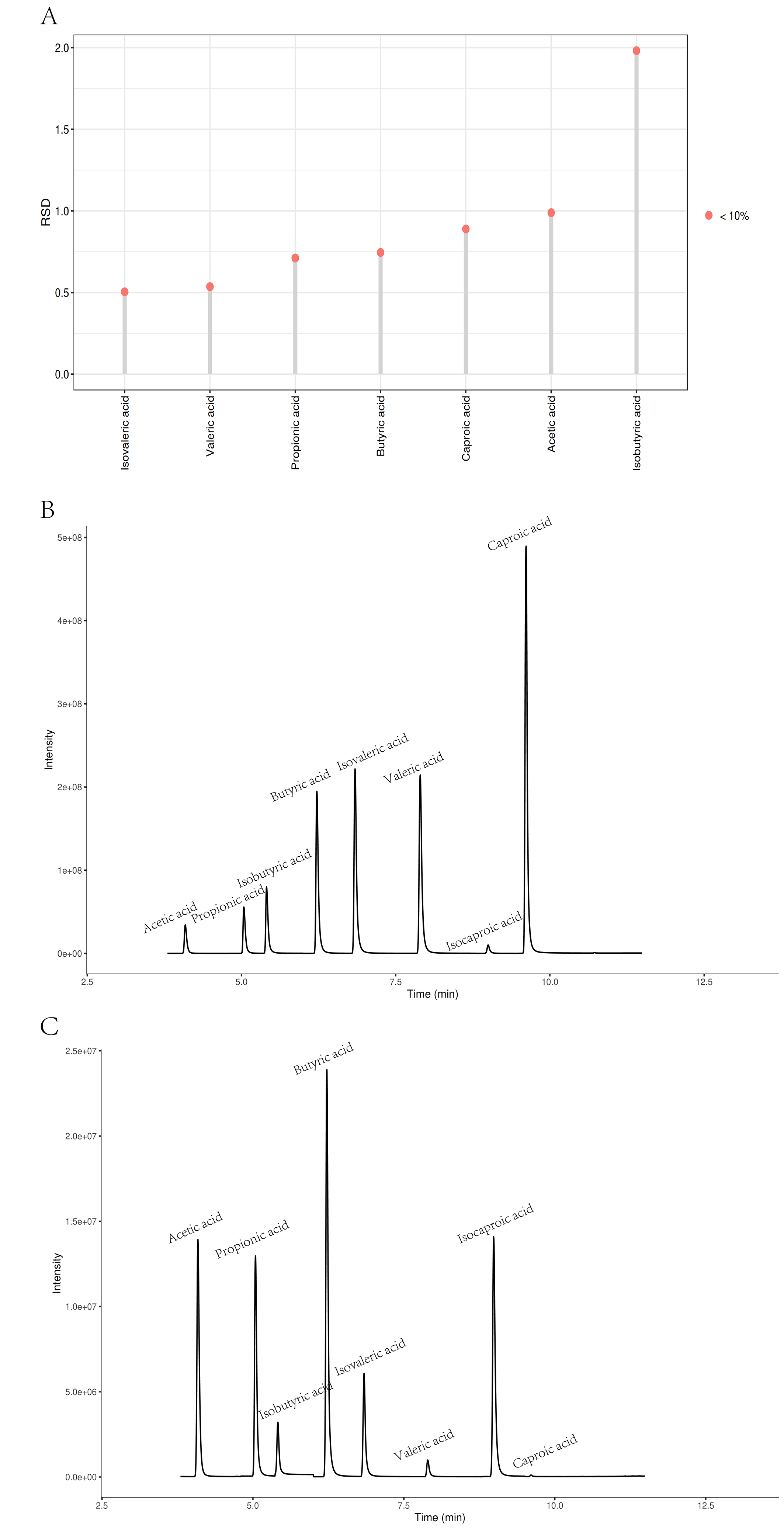

Supplement: FIGURE S1 — Stability of short-chain fatty acids in QC samples, mixed sample and sample TIC plots. (A) Stability of short-chain fatty acids in QC samples; (B) mixed-label TIC plot; (C) sample TIC plot. [file Image_1.TIF]
