# Supplementary material for: Integrated 16S rDNA-Seq and metabolomics reveal seasonal dynamics of gut microbial–SCFA–immune crosstalk in diarrheic calves
Source: Front Vet Sci. 2025 Jul 11;12:1615310. doi: 10.3389/fvets.2025.1615310 (PMC12290471; doi:10.3389/fvets.2025.1615310)
Supplement: Supplementary file 2 [file Table_1.docx]

**Table S1**

Linear regression equations for GC-MS standard sample detection

| **SCFA** | **RT** | **Calibration curve** | **R2** | **Linearity range (μg/mL)** |
| --- | --- | --- | --- | --- |
| Acetic acid | 4.68 | Y = 0.003818*x + 0.014 | 0.9989 | 0.02 - 500.0 |
| Propionic acid | 5.75 | y = 0.006508*x + 0.0004944 | 0.9967 | 0.02 - 500.0 |
| Isobutyric acid | 6.15 | Y = 0.0111*x + 0.0002129 | 0.9977 | 0.02 - 500.0 |
| Butyric acid | 7.04 | y = 0.02403*x + 0.0007154 | 0.9969 | 0.02 - 500.0 |
| Isovaleric acid | 7.7 | Y = 0.02918*x + 0.000622 | 0.9959 | 0.02 - 500.0 |
| Valeric acid | 8.84 | y = 0.03169*x + 0.0001289 | 0.9944 | 0.02 - 500.0 |
| Caproic acid | 10.27 | Y = 0.1038*x + 0.00311 | 0.9951 | 0.02 - 250.0 |
